# Supplementary material for: Improving and Streamlining Gene Editing in Yarrowia lipolytica via Integration of Engineered Cas9 Protein
Source: J Fungi (Basel). 2024 Jan 12;10(1):63. doi: 10.3390/jof10010063 (PMC10817246; doi:10.3390/jof10010063)
Supplement: Supplementary file 1 [file jof-10-00063-s001.zip › Table S1.pdf]

Table S1. Codon-optimized sequence of *eSpCas9* gene

Gene	Sequence(5’-3’)
Codon-optimized	GACAAGAAGTACTCAATCGGCCTGGACATTGGCACCAACTCTGTTGGTT
eSpCas9	GGGCCGTGATTACCGACGAATACAAAGTGCCCTCTAAAAAGTTCAAGG
	TCCTTGGCAACACAGATCGACATTCGATCAAGAAGAACCTGATAGGAG
	CACTGCTCTTCGACTCGGGTGAGACTGCGGAGGCCACCCGGCTGAAAC
	GAACCGCCCGACGACGTTACACTCGCAGAAAGAACAGAATTTGTTACT
	TGCAGGAAATCTTCTCGAACGAGATGGCCAAGGTGGACGACTCGTTCT
	TCCACCGACTTGAGGAGTCCTTCCTGGTTGAGGAGGACAAGAAACACG
	AGCGTCATCCCATTTTTGGAAACATTGTCGACGAGGTCGCTTACCACGA
	AAAGTATCCAACAATCTACCATCTGCGCAAGAAGCTGGTAGACTCAACC
	GACAAGGCAGATCTGCGGCTTATCTATCTGGCGCTGGCCCACATGATTA
	AGTTCAGAGGCCATTTCCTGATTGAGGGTGATCTCAACCCAGACAACTC
	TGATGTCGACAAGCTGTTCATTCAGCTGGTGCAGACCTACAACCAGCTG
	TTTGAAGAGAACCCCATCAATGCCTCAGGTGTCGATGCCAAAGCCATTC
	TGTCCGCGCGACTCTCTAAGAGCCGACGGCTAGAGAATCTGATCGCACA
	ACTTCCAGGAGAGAAGAAGAATGGATTATTTGGAAACCTGATCGCCCTT
	TCGCTAGGCTTGACGCCCAACTTCAAGTCTAACTTTGATCTAGCGGAGG
	ACGCCAAGCTGCAGCTTTCTAAGGACACATACGACGATGATCTAGACAA
	TCTGCTTGCGCAGATCGGTGACCAGTACGCTGATTTGTTTTTAGCTGCCA
	AGAACTTGAGCGACGCTATCCTCCTCTCCGACATCCTACGGGTGAACAC
	CGAGATTACTAAGGCCCCCTTGTCAGCTTCGATGATCAAGCGGTACGAT
	GAACATCACCAGGACCTGACACTACTCAAGGCACTCGTGCGCCAGCAG
	CTTCCTGAAAAGTATAAGGAAATTTTCTTTGACCAAAGCAAAAATGGTT
	ACGCCGGCTACATTGATGGTGGTGCATCTCAGGAGGAGTTCTACAAGTT
	TATCAAGCCGATTCTCGAGAAAATGGACGGAACCGAGGAACTCCTTGT
	CAAGCTCAACCGAGAAGATCTACTGCGTAAGCAGAGAACCTTTGACAA
	TGGATCCATTCCTCATCAGATCCACCTCGGCGAACTGCATGCCATCCTCC
	GTCGACAGGAGGACTTCTATCCATTTCTGAAAGACAACCGGGAAAAAA
	TTGAAAAGATTCTTACGTTCCGAATTCCCTACTACGTTGGACCTCTGGCT
	CGAGGAAATTCCCGATTCGCCTGGATGACTAGAAAGTCTGAGGAGACC
	ATCACCCCCTGGAACTTTGAGGAGGTGGTGGACAAGGGAGCCTCTGCC
	CAGTCCTTTATCGAGCGAATGACCAACTTCGACAAAAATCTGCCCAATG
	AGAAGGTGCTGCCCAAGCACTCTCTGTTGTATGAGTACTTCACCGTCTA
	CAACGAGCTCACCAAGGTTAAGTATGTGACCGAAGGAATGCGTAAGCC
	TGCGTTTTTATCGGGTGAGCAAAAGAAGGCGATCGTCGATCTGCTGTTT
	AAGACCAACCGCAAGGTCACAGTCAAACAACTGAAGGAGGATTATTTC
	AAGAAGATCGAGTGCTTCGACAGTGTTGAGATCTCGGGTGTGGAGGAC
	CGGTTCAACGCCTCACTTGGAACTTATCACGATCTCCTGAAGATCATCA
	AGGATAAGGACTTTCTGGACAACGAAGAGAATGAGGACATCCTGGAAG
	ATATTGTGTTGACTCTCACTTTGTTCGAGGACAGGGAAATGATTGAAGA
	ACGTCTCAAGACCTACGCACACCTGTTCGACGACAAGGTCATGAAACA
	GCTAAAACGGCGACGATACACTGGCTGGGGACGACTCTCGCGTAAGTT

AATCAACGGCATCCGAGATAAACAGAGCGGCAAGACGATCCTGGATTT
CCTTAAGTCGGATGGATTCGCCAACCGAAACTTCATGCAGCTCATTCAC
GACGACTCACTCACTTTTAAGGAAGACATTCAGAAGGCTCAGGTTTCTG
GACAGGGCGACTCCCTTCATGAGCACATTGCTAACCTCGCGGGGTCGCC
TGCAATCAAGAAGGGTATTCTCCAGACCGTTAAGGTGGTTGATGAGCTG
GTCAAGGTGATGGGCCGACACAAGCCCGAGAACATCGTTATTGAGATG
GCTCGAGAGAACCAGACAACCCAGAAGGGCCAGAAGAATTCTCGTGA
GAGAATGAAGCGAATTGAGGAGGGTATTAAGGAGCTAGGGTCCCAGAT
TCTCAAGGAACACCCGGTTGAGAATACTCAACTCCAAAACGAGAAGCT
GTACCTTTACTACCTGCAGAATGGTCGGGACATGTACGTCGATCAGGAG
CTTGATATCAACCGACTGAGCGACTATGACGTGGACCACATTGTTCCGC
AGTCTTTTCTCGCTGACGACTCCATAGATAACAAAGTTTTGACTCGATCC
GATAAGAACCGAGGAAAGTCCGATAACGTGCCTAGCGAAGAAGTCGTG
AAGAAGATGAAGAACTACTGGCGACAGCTCTTGAATGCTAAGCTCATTA
CTCAGCGGAAATTCGACAATCTTACTAAGGCCGAGCGAGGCGGACTTTC
CGAACTCGACAAGGCAGGCTTCATTAAGCGACAATTGGTAGAGACGCG
ACAAATCACCAAGCATGTGGCCCAAATTCTCGACTCGAGGATGAACAC
AAAGTACGATGAGAACGACAAACTTATCCGAGAAGTGAAGGTGATAAC
CCTTAAGTCCAAGCTTGTCTCCGACTTCCGAAAGGATTTCCAGTTTTAC
AAGGTGCGGGAGATCAATAACTATCACCACGCTCATGATGCCTACCTGA
ACGCAGTTGTGGGTACTGCCCTCATTAAAAAGTACCCTGCTCTTGAGAG
TGAGTTTGTCTATGGAGACTACAAGGTCTACGACGTCAGAAAGATGATT
GCTAAATCTGAGCAGGAGATCGGCAAGGCGACAGCTAAATACTTTTTCT
ACTCCAACATCATGAACTTCTTCAAGACTGAGATCACCCTAGCCAACGG
CGAGATCAGAAAGGCTCCTCTGATCGAGACGAATGGAGAGACAGGTGA
GATTGTGTGGGACAAGGGCAGAGACTTTGCCACTGTGCGAAAGGTCCT
GAGCATGCCCCAGGTCAACATTGTAAAAAAGACCGAGGTTCAGACGGG
GGGGTTCTCTAAGGAAAGTATTCTGCCGAAGAGGAACTCTGACAAGTT
AATCGCACGCAAAAAGGACTGGGACCCCAAGAAGTATGGAGGATTTGA
TTCCCCTACGGTTGCTTACAGTGTGTTGGTGGTCGCAAAAGTCGAGAAG
GGCAAGTCGAAGAAACTCAAGAGTGTGAAGGAGCTTCTGGGGATTACT
ATCATGGAGAGATCGTCTTTTGAGAAGAACCCTATCGACTTCCTAGAGG
CTAAGGGCTACAAGGAGGTCAAGAAAGACCTGATTATTAAGCTCCCCA
AGTACTCCCTGTTCGAGCTGGAGAACGGACGAAAGAGGATGCTGGCCA
GCGCCGGAGAATTACAAAAGGGCAACGAACTGGCCCTGCCTTCTAAAT
ATGTCAACTTTTTGTACCTTGCTTCCCATTACGAGAAACTTAAGGGCTCT
CCAGAGGATAACGAGCAGAAACAACTCTTTGTGGAGCAGCATAAACAC
TACCTGGACGAGATCATTGAGCAAATTTCCGAGTTCTCCAAAAGAGTCA
TTCTGGCCGATGCTAACCTCGACAAGGTTCTGTCTGCGTACAACAAGCA
CAGAGATAAGCCCATCAGAGAACAGGCCGAAAACATCATTCATTTGTTC
ACACTTACAAACCTGGGTGCTCCCGCTGCTTTTAAGTACTTTGACACCA
CCATTGACCGTAAGCGCTATACATCAACTAAGGAAGTTCTCGATGCCAC
GCTGATCCACCAGTCAATCACCGGTTTGTACGAAACCCGCATTGACCTG
AGTCAGCTCGGTGGCGAT
